# *Legionella* pneumonia due to non-*Legionella pneumophila* serogroup 1: usefulness of the six-point scoring system

**DOI:** 10.1186/s12890-017-0559-3

**Published:** 2017-12-16

**Authors:** Akihiro Ito, Tadashi Ishida, Yasuyoshi Washio, Akio Yamazaki, Hiromasa Tachibana

**Affiliations:** 1 Department of Respiratory Medicine, Ohara Healthcare Foundation, Kurashiki Central Hospital, Miwa 1-1-1, Kurashiki, Okayama, 710-8602 Japan; 2 Department of Respiratory Medicine, Saiseikai Fukuoka General Hospital, Tenjinn 1-3-46, Chuoku, Fukuoka, Fukuoka 810-0001 Japan; 3 Department of Respiratory Medicine, National Hospital Organization Minami Kyoto Hospital, Nakaashihara 11, Joyo, Kyoto, 610-0113 Japan

**Keywords:** *Legionella* pneumonia, *Legionella pneumophila* serogroup 1, Community-acquired pneumonia, Diagnostic scoring system

## Abstract

**Background:**

Because of a limited number of reports, we aimed to investigate the clinical characteristics of patients with *Legionella* pneumonia due to non-*Legionella pneumophila* serogroup 1 and the diagnostic usefulness of the six-point scoring system for such patients compared with patients with pneumonia caused by *L. pneumophila* serogroup 1.

**Methods:**

We retrospectively analysed patients diagnosed with *Legionella* pneumonia due to non-*L. pneumophila* serogroup 1 between March 2001 and June 2016. We examined the clinical characteristics, including symptoms, laboratory findings, radiologic findings, pneumonia severity, initial treatment and prognosis. We also calculated scores using the six-point scoring system in these patients. Furthermore, we compared the clinical characteristics and six-point scores between non-*L. pneumophila* serogroup 1 patients and *L. pneumophila* serogroup 1 patients among hospitalized community-acquired pneumonia patients enrolled prospectively between October 2010 and July 2016.

**Results:**

Eleven patients had pneumonia due to non-*L. pneumophila* serogroup 1; their median age was 66 years and 8 patients (72.7%) were male. The most common pathogen was *L. pneumophila* serogroup 3 (6/11), followed by *L. pneumophila* serogroup 9 (3/11), *L. pneumophila* serogroup 6 (1/11) and *L. longbeachae* (1/11). Non-specific symptoms, such as fever and cough, were common. Six patients (54.5%) had liver enzyme elevation, but no patient developed hyponatraemia at <130 mEq/L. Nine patients (81.8%) showed lobar pneumonia and 7 patients (63.6%) manifested with consolidation and ground-glass opacity. Patients with mild to moderate severity comprised 10 (90.9%) by CURB-65 and 5 (45.5%) by the Pneumonia Severity Index. Of all patients, 4 were admitted to the intensive care unit and 3 died despite appropriate empiric therapy. The clinical characteristics were not significantly different between non-*L. pneumophila* serogroup 1 patients and *L. pneumophila* serogroup 1 patients (*n* = 23). At a cut-off value of ≥ 2 points, the sensitivity of the six-point scoring system was 54.5% (6/11) for non-*L. pneumophila* serogroup 1 patients and 95.7% (22/23) for *L. pneumophila* serogroup 1 patients.

**Conclusions:**

Cases of non-*L. pneumophila* serogroup 1 pneumonia varied in severity from mild to severe and the clinical characteristics were often non-specific. The six-point scoring system was not useful in predicting such *Legionella* pneumonia cases.

**Electronic supplementary material:**

The online version of this article (10.1186/s12890-017-0559-3) contains supplementary material, which is available to authorized users.

## Background

*Legionella* pneumonia is a type of pulmonary infection that is caused by Gram-negative bacilli and is an important cause of severe community-acquired pneumonia (CAP) [1]. The most frequently identified causative pathogen of *Legionella* pneumonia is *Legionella pneumophila* serogroup 1 [2, 3], probably because it can be quickly diagnosed by the urinary antigen test. However, about 20% of *Legionella* pneumonia cases due to non-*L. pneumophila* serogroup 1 are diagnosed not by the urinary antigen test, but by culture using Wadowsky-Yee-Okuda (WYO)-α or Buffered Charcoal Yeast Extract (BCYE)-α medium [2, 3]. *Legionella* pneumonia due to non-*L. pneumophila* serogroup 1 can be both severe [4–8] and mild to moderate [9, 10].

To date, there have been few reports that summarised the clinical characteristics of such cases, including symptoms, laboratory findings, radiologic findings, pneumonia severity, treatment and prognosis. To predict the probability of *Legionella* pneumonia, Fiumefreddo et al. [11] proposed a six-point scoring system using dichotomised routine clinical and laboratory variables. Haubitz et al. [12] validated the usefulness of this scoring system using a large multinational database. However, to our knowledge, there are no reports evaluating the usefulness of this scoring system in predicting non-*L. pneumophila* serogroup 1 *Legionella* pneumonia. This study aimed to investigate the clinical characteristics of *Legionella* pneumonia due to non-*L. pneumophila* serogroup 1 and the usefulness of the six-point scoring system in predicting such cases compared with those of *L. pneumophila* serogroup 1.

## Methods

### Study design and setting

This study retrospectively analysed hospitalised and outpatient CAP cases due to *Legionella* species other than *L. pneumophila* serogroup 1 at Kurashiki Central Hospital between March 2001 and June 2016. CAP was diagnosed in accordance with the latest Infectious Diseases Society of America/American Thoracic Society guidelines [13] as follows: at least one clinical symptom (fever, cough, sputum production, dyspnoea or pleuritic chest pain) plus at least more than one finding of elevated inflammatory biomarkers or coarse crackles on auscultation, in addition to new infiltrate on chest radiography. Exclusion criteria were age < 15 years, acquired immune deficiency syndrome, hospital-acquired pneumonia and healthcare-associated pneumonia [14]. A total of 11 patients with *Legionella* pneumonia due to non-*L. pneumophila* serogroup 1 were finally included in this analysis. This study was approved by the institutional review board of Kurashiki Central Hospital (approval number 2364). Based on the Ethical Guidelines for Medical and Health Research Involving Human Subjects of the Ministry of Health, Labour and Welfare, we notify the research subjects of, or make public, information concerning the research on the web. All patients gave their informed consent to participate in this study by being given opportunities to refuse to participate.

In all patients, the severity of pneumonia was assessed on admission or on the first visit using the Pneumonia Severity Index (PSI) [15] and CURB-65 score [confusion, urea > 7 mmol/L, respiratory rate ≥ 30 breaths/min, low blood pressure (systolic < 90 mmHg or diastolic ≤ 60 mmHg) and age ≥ 65 years)] [16]. All patients were administered antimicrobial agents based on the decision of the physician in charge and in accordance with the recommendations of the CAP guidelines of the Japanese Respiratory Society [17]. Blood tests and chest X-ray images were examined to assess the effectiveness of the antimicrobials as appropriate. We treated patients in the intensive care unit (ICU) if they needed mechanical ventilatory support and/or vasopressor drugs.

In all patients, the variables examined included age, sex, comorbidity, smoking history, symptoms, bacterial strain, chest X-ray or computed tomography (CT) findings, laboratory findings, CURB-65 and PSI severity scores, initial treatment and prognosis.

### Microbiologic investigation

On admission, sputum and blood for cultures, serum for measuring antibodies and urine for *Streptococcus pneumoniae* or *L. pneumophila* antigen test were collected to detect as clearly as possible the causative microorganisms of CAP. *Legionella* species was identified using culture on WYO-α medium, not by polymerase chain reaction. We diagnosed *Legionella* pneumonia due to non-*L. pneumophila* serogroup 1 based on sputum or blood culture.

### Radiologic findings

Radiologic tests were examined by chest X-ray and/or CT, which were interpreted by four pulmonologists. We examined the affected lobes, radiologic pattern and the presence of consolidation, ground-glass opacity (GGO), nodule, pleural effusion, cavity or lymphadenopathy. We defined airspace consolidation as infiltration with obscured vascular margins and GGO as an increase in hazy attenuation with intact vascular markings. Mediastinal lymphadenopathy was defined as the presence of lymph nodes greater than 10 mm in minimal diameter.

### Clinical characteristics of *Legionella* pneumonia due to non-*L. pneumophila* serogroup 1 and *L. pneumophila* serogroup 1

We also investigated clinical characteristics including age, sex, smoking history, vital signs, laboratory findings and pneumonia severity in *Legionella* pneumonia due to *L. pneumophila* serogroup 1 patients and compared them with those of non-*L. pneumophila* serogroup 1 patients. *L. pneumophila* serogroup 1 patients were diagnosed by the urinary antigen test and/or sputum culture or serum antibody. These patients were part of a prospective, observational cohort at our hospital between October 2010 and July 2016 (UMIN000004353). All patients with *Legionella* pneumonia due to *L. pneumophila* serogroup 1 also gave their informed consent to participate in this study.

### The six-point scoring system for the diagnosis of *Legionella* pneumonia

The six-point scoring system was determined in each case and comprised dichotomised routine clinical and laboratory variables, including fever >39.4 °C, C-reactive protein (CRP) value >187 mg/L, lactate dehydrogenase >225 mmol/L, thrombocytopenia <171 × 10^9^/L, hyponatraemia (serum sodium <133 mmol/L) and unproductive cough. The six-point scores of non-*L. pneumophila* serogroup 1 patients were compared with those of *L. pneumophila* serogroup 1 patients.

### Statistical analysis

Categorical variables are expressed as frequency (percentage) and continuous variables are expressed as medians and interquartile ranges (IQR). Categorical variables were analysed using Fisher’s exact test and continuous variables were analysed by the non-parametric Mann–Whitney U-test. A *P* value of < 0.05 was considered significant. Statistical analyses were performed using R (version 3.0.3, Vienna, Austria).

## Results

### Patients’ characteristics

The characteristics of the study population are shown in Table 1. Cases 1, 2, 6 and 9 were outpatients and the others were inpatients. The age range was 58–82 years. Eight patients (72.7%) were male. Diabetes mellitus, chronic liver disease and malignant disease were seen in 2 patients (18.2%) each and 6 patients (54.5%) had a smoking history (3 were past, 3 were current). The most common symptom was fever (72.7%), followed by cough (54.5%) and sputum production (54.5%). Disturbance in consciousness was seen in only 1 patient; no patient had digestive symptoms, such as abdominal pain, vomiting and diarrhoea. The most common bacterial strain was *L. pneumophila* serogroup 3 (54.5%), followed by *L. pneumophila* serogroup 9 (27.3%), *L. pneumophila* serogroup 6 (9.1%) and *L. longbeachae* (9.1%).

**Table 1:** Characteristics of all patients with *Legionella* pneumonia due to non-*Legionella pneumophila* serogroup 1

	Age (y)	Sex	Comorbidity	Smoking history	Symptoms	*Legionella* species
1	58	F	None	Never	Cough, sputum	*L. pneumophila* SG3
2	58	F	RA, steroid use	Unknown	Cough, sputum	*L. pneumophila* SG3
3	59	M	DM, CLD, heavy drinker	Current	Fever, consciousness disturbance	*L. pneumophila* SG9
4	60	F	Malignant disease	Unknown	Fever	*L. pneumophila* SG3
5	65	M	None	Past	Fever, myalgia, arthralgia	*L. pneumophila* SG9
6	66	M	DM, CLD	Past	Fever, cough, sputum	*L. pneumophila* SG3
7	68	M	None	Current	Fever, dyspnoea	*L. longbeachae*
8	71	M	None	Unknown	Fever, cough, sputum, arthralgia	*L. pneumophila* SG6
9	74	M	Malignant disease	Past	Fever, cough, sputum, haemoptysis, dyspnoea	*L. pneumophila* SG3
10	77	M	COPD, asthma	Past	Dyspnoea	*L. pneumophila* SG3
11	82	M	None	Unknown	Fever, cough, sputum, dyspnoea	*L. pneumophila* SG9

*Abbreviations*: *CLD* chronic liver disease, *COPD* chronic obstructive pulmonary disease, *DM* diabetes mellitus, *RA* rheumatoid arthritis, *SG* serogroup

### Radiologic findings

Table 2 shows the chest X-ray and CT findings. Chest CT scan was available for all patients except one. There were 4 patients (Cases 3, 5, 8 and 9) with unilobar infiltration and 7 patients (Cases 1, 2, 4, 6, 7, 10 and 11) with multilobar infiltration. All patients, except two (Cases 1 and 2) (81.8%), had lobar pneumonia. The most common finding was GGO (81.8%), followed by consolidation (72.7%). Consolidation plus GGO was seen in 7 patients (63.6%). Nodule and pleural effusion were found in 3 patients (27.3%) each; there was no cavitary lesion in any of the patients.

**Table 2:** Chest imaging findings in all patients with *Legionella* pneumonia due to non-*Legionella pneumophila* serogroup 1

	Modality	Affected Lobes	Radiologic pattern	Consolidation	GGO	Nodule	Effusion	Cavity	Lymph node swelling
1	CT	LUL, LLL	Lobular	–	–	+	–	–	+
2	CT	RUL, RML, LUL	Lobular	–	+	+	–	–	–
3	CT	LUL	Lobar	+	+	–	+	–	–
4	CT	RUL, RML, RLL, LUL, LLL	Lobar	+	+	–	–	–	–
5	Xp	LLL	Lobar	+	+	–	–	–	–
6	CT	RUL, RLL, LLL	Lobar	–	+	+	–	–	–
7	CT	RUL, RLL, LUL, LLL	Lobar	+	+	–	+	–	+
8	CT	LUL	Lobar	+	–	–	–	–	–
9	CT	RUL	Lobar	+	+	–	+	–	+
10	CT	LUL, LLL	Lobar	+	+	–	–	–	–
11	CT	RUL, RML, RLL, LUL, LLL	Lobar	+	+	–	–	–	–

*Abbreviations*: *CT* computed tomography, *GGO* ground-glass opacity, *LLL* left lower lobe, *LUL* left upper lobe, *RLL* right lower lobe, *RML* right middle lobe, *RUL* right upper lobe, *Xp* radiograph

### Laboratory findings

Table 3 shows the laboratory findings in all non-*L. pneumophila* serogroup 1 patients. High CRP concentration was found in 9 patients (81.8%); a white blood cell count > 10 × 10^3^/μL was seen in only 3 patients (27.3%). Elevated liver enzyme, either aspartate aminotransferase or alanine aminotransferase, was seen in 6 patients (54.5%). Hyponatraemia was seen in only 3 patients (27.3%). Among 4 patients in whom creatine phosphokinase (CPK) was measured, 2 (50%) had high values.

**Table 3:** Laboratory findings in all patients with *Legionella* pneumonia due to non-*Legionella pneumophila* serogroup 1

	WBC (×10^3^/μL)	CRP (mg/L)	TP (g/dL)	Alb (g/dL)	AST (IU/L)	ALT (IU/L)	LDH (IU/L)	BUN (mg/dL)	Cr (mg/dL)	Na (mEq/L)	Plt (×10^9^/L)	CPK (IU/L)
1	6.6	1.1	8.3	4.4	21	13	195	15	0.67	139	304	ND
2	8.8	0.6	6.2	3.6	32	35	214	23	0.80	141	226	ND
3	7.3	328.6	6.2	2.5	636	274	1309	23	1.5	131	197	9757
4	5.9	174.7	3.3	1.3	33	20	421	51	2.74	142	13	ND
5	8.2	195.1	5.9	2.7	49	36	256	13	0.80	130	202	167
6	6.9	222	7.3	2.9	30	45	204	15	0.95	136	261	ND
7	14.8	345.4	5.1	2.2	50	29	381	14	0.69	133	215	61
8	10.2	125	6.7	2.7	17	4	314	12	0.7	137	323	ND
9	8.3	266.3	5.8	2.5	22	20	117	12	0.82	131	120	ND
10	9.6	5.6	6.9	3.6	24	21	198	13	0.85	141	190	ND
11	14.1	282	7.1	3.5	190	60	885	34	1.6	137	132	6728

*Abbreviations*: *Alb* albumin, *ALT* alanine aminotransferase, *AST* aspartate aminotransferase, *BUN* blood urea nitrogen, *CPK* creatine phosphokinase, *Cr* creatinine, *CRP* C-reactive protein, *LDH* lactate dehydrogenase, *ND* not done, *Plt* platelet, *TP* total protein, *WBC* white blood cell

### Severity of pneumonia, initial treatment and prognosis

Table 4 shows the pneumonia severity, initial treatment and prognosis in all non-*L. pneumophila* serogroup 1 patients. Most patients (10/11, 90.9%) had mild to moderate pneumonia by CURB-65 (≤ 2 points) and about half (5/11, 45.5%) had mild to moderate pneumonia by PSI (≤class III). Four patients (36.4%) were treated in the ICU. All patients were administered empiric antimicrobials that covered *Legionella* species; however, 3 patients (27.3%) died.

**Table 4:** Severity of pneumonia, treatment and prognosis in all non-*Legionella pneumophila* serogroup 1 patients

	CURB-65 (points)	PSI (class)	Initial treatment	ICU admission	Outcome
1	0	II	AZM-PO	–	Survived
2	0	II	AZM-PO	–	Survived
3	2	IV	LVFX-IV, AZM-IV	+	Survived
4	1	IV	MEPM, CPFX-IV	+	Died
5	2	IV	CTRX, AZM-PO	–	Survived
6	1	III	AZM-PO	–	Survived
7	2	IV	CTRX, LVFX-IV	+	Died
8	1	III	LVFX-PO	–	Survived
9	1	IV	LVFX-PO	–	Survived
10	2	III	LVFX-PO	–	Survived
11	4	V	MEPM, CPFX-IV	+	Died

*Abbreviations*: *AZM* azithromycin, *CPFX* ciprofloxacin, *CTRX* ceftriaxone, *CURB-65* confusion, urea > 7 mmol/L, respiratory rate ≥ 30 breaths/min, low blood pressure (systolic < 90 mmHg or diastolic ≤ 60 mmHg) and age ≥ 65 years, *ICU* intensive care unit, *IV* intravenous, *LVFX* levofloxacin, *MEPM* meropenem, *PO* per os, *PSI* Pneumonia Severity Index

### Clinical characteristics of non-*L. pneumophila* serogroup 1 and *L. pneumophila* serogroup 1 patients

Between October 2010 and July 2016, we prospectively enrolled 1236 hospitalized CAP patients, of which 23 patients had *Legionella* pneumonia due to *L. pneumophila* serogroup 1. Eleven patients were diagnosed by using the urinary antigen test and sputum culture, 11 patients with only the urinary antigen test and 1 patient with serum antibody. Clinical characteristics including age, sex, comorbidities, vital signs, laboratory examinations and severity scores between non-*L. pneumophila* serogroup 1 patients and *L. pneumophila* serogroup 1 patients were not significantly different (Table 5). The frequency of ICU admission was not significantly different between these two groups, however, in-hospital mortality was significantly higher in non-*L. pneumophila* serogroup 1 patients than *L. pneumophila* serogroup 1 patients (27.3% vs 0%, *P* = 0.03).

**Table 5:** Clinical characteristics of non-*Legionella pneumophila* serogroup 1 and *Legionella pneumophila* serogroup 1 patients

	non-*L. pneumophila* serogroup 1 (*n* = 11)	*L. pneumophila* serogroup 1 (n = 23)	*P* value
Age (y)	66.0 (59.5–72.5)	70.0 (58.5–74.5)	0.96
Male	8 (72.7)	21 (91.3)	0.30
Smoking history			
Current + Past	5 (62.5)	17 (73.9)	0.66
Comorbidity			
Chronic heart disease	0 (0)	7 (30.4)	0.07
COPD	1 (9.1)	0 (0)	0.32
Diabetes mellitus	2 (18.2)	7 (30.4)	0.68
Cerebrovascular disease	0 (0)	5 (21.7)	0.15
Malignant disease	2 (18.2)	2 (8.7)	0.58
Chronic kidney disease	0 (0)	3 (13.0)	0.54
Chronic liver disease	2 (18.2)	0 (0)	0.1
Vital signs			
Body temperature (°C)	37.7 (36.9–38.6)	38.8 (37.8–39.5)	0.06
Systolic blood pressure (mmHg)	121 (121–130)	136 (122–157)	0.11
Heart rate (beats/min)	95 (86–117)	100 (87–104)	0.82
Laboratory examinations			
Alb (g/dL)	2.7 (2.5–3.6)	3.0 (2.3–3.3)	0.66
LDH (U/L)	256 (201–401)	395 (276–540)	0.12
BUN (mg/dL)	15 (13–23)	24 (16–41)	0.14
Cr (mg/dL)	0.82 (0.75–1.23)	1.06 (0.83–1.74)	0.12
Na (mmol/L)	137 (132–140)	134 (130–137)	0.14
Ht (%)	39.7 (33.2–42.1)	38.9 (35.2–41.8)	0.90
Plt (×10^4^/μL)	20.2 (16.1–24.4)	16.4 (14.2–19.8)	0.16
WBC (×10^3^/μL)	8.3 (7.1–9.9)	10.4 (8.5–11.9)	0.11
CRP (mg/L)	195 (65–274)	230 (184–281)	0.31
CURB-65 (score)			0.48
0	2 (18.2)	2 (8.7)	
1	4 (36.4)	7 (30.4)	
2	4 (36.4)	8 (34.8)	
3	0 (0)	5 (21.7)	
4	1 (9.1)	1 (4.3)	
5	0 (0)	0 (0)	
PSI (score)	99 (79–116)	97 (84–121)	0.73
PSI (class)			0.84
I	0 (0)	1 (4.3)	
I	2 (18.2)	2 (8.7)	
III	3 (27.3)	6 (26.1)	
IV	5 (45.5)	9 (39.1)	
V	1 (9.1)	5 (21.7)	
ICU admission	4 (36.4)	6 (26.1)	0.69
In-hospital mortality	3 (27.3)	0 (0)	0.03

Data are presented as medians (interquartile range) or n (%)

*Abbreviations*: *Alb* albumin, *BUN* blood urea nitrogen, *COPD* chronic obstructive pulmonary disease, *Cr* creatinine, *CRP* C-reactive protein, *CURB-65* confusion, urea > 7 mmol/L, respiratory rate ≥ 30 breaths/min, low blood pressure (systolic < 90 mmHg or diastolic ≤ 60 mmHg) and age ≥ 65 years, *Ht* haematocrit, *ICU* intensive care unit, *LDH* lactate dehydrogenase, *Na* sodium, *Plt* platelet, *PSI* Pneumonia Severity Index, *WBC* white blood cell

### The six-point scoring system for predicting *Legionella* pneumonia

The six-point scores for predicting *Legionella* pneumonia due to non-*L. pneumophila* serogroup 1 are shown in Table 6 and those for predicting *Legionella* pneumonia due to *L. pneumophila* serogroup 1 are shown in Additional file 1: Table S1. The median six-point score was significantly lower in non-*L. pneumophila* serogroup 1 patients than in *L. pneumophila* serogroup 1 patients (2.0, IQR 0.5–3.0 vs. 3.0, IQR 2.0–3.5; *P* = 0.021). At a cut-off value of ≥2 points, the sensitivity of this scoring system was 54.5% (6/11) for non-*L. pneumophila* serogroup 1 patients and 95.7% (22/23) for *L. pneumophila* serogroup 1 patients.

**Table 6:** The six-point scoring system for predicting *Legionella* pneumonia in non-*Legionella pneumophila* serogroup 1 patients

	CRP > 187 mg/L	Na < 133 mmol/L	Temperature^a^ > 39.4 °C (°C)	Plt < 171 × 10^9^/L	LDH > 225 IU/L	Dry cough	Total score
1	–	–	- (37.0)	–	–	–	0
2	–	–	- (36.5)	–	–	–	0
3	+	+	- (39.2)	–	+	–	3
4	–	–	- (38.8)	+	+	–	2
5	+	+	- (37.7)	–	+	–	3
6	+	–	- (36.7)	–	–	–	1
7	+	–	- (38.3)	–	+	–	2
8	–	–	- (38.3)	–	+	–	1
9	+	+	- (37.0)	+	–	–	3
10	–	–	- (36.5)	–	–	–	0
11	+	–	- (39.3)	+	+	–	3

*Abbreviations*: *CRP* C-reactive protein, *LDH* lactate dehydrogenase, *Na* sodium, *Plt* platelet

^a^The patient’s body temperature is indicated in parentheses

## Discussion

This study on 11 patients demonstrated the clinical characteristics of *Legionella* pneumonia due to non-*L. pneumophila* serogroup 1. *Legionella* pneumonia is a principal cause of severe CAP [18], particularly among patients < 60 years, in whom the rate of severe pneumonia due to *Legionella* species was reported to be as high as 14.1% compared with 2.3% in patients ≥ 60 years [1]. In this study, almost all patients had *Legionella* pneumonia of mild to moderate severity by CURB-65, and 4 cases could be treated as outpatients; however, 4 patients were admitted to the ICU and 3 of those died despite appropriate empiric therapy.

The reported comorbidities that could predispose one to *Legionella* pneumonia were chronic lung disease [19], glucocorticoid treatment [20], haematologic malignancies under chemotherapy [21] and solid tumours [22]. In this study, 1 patient had chronic obstructive pulmonary disease, 1 received steroid treatment and 2 had malignant disease; however, almost half of the patients (5/11, 45.5%) had no comorbidities.

Cunha et al. reported that *Legionella* pneumonia presented with non-specific symptoms of fever > 38.8 °C in 67–100%, cough in 41–92%, chills in 15–77% and dyspnoea in 36–56%, as well as relatively specific symptoms of neurologic abnormalities in 38–53%, myalgia or arthralgia in 20–43%, diarrhoea in 19–47%, chest pain in 14–50%, headache in 17–43% and nausea or vomiting in 9–25% [23]. In our case series of *Legionella* pneumonia, many patients manifested with fever (8/11, 72.7%) and cough (6/11, 54.5%), but only a few had arthralgia or myalgia (2/11, 18.2%) and disturbance in consciousness (1/11, 9.1%). No patient had specific symptoms of diarrhoea, nausea or vomiting.

For laboratory findings, hepatic dysfunction, hyponatraemia [24] and CPK elevation [25] were indicated as more significant findings in *Legionella* pneumonia patients than in those with pneumonia from other aetiology. Almost half of our patients (5/11, 45.5%) had normal liver enzymes, no patient had serum sodium < 130 mEq/L and only 2 of 4 had CPK elevation. The number of patients who did not have liver enzyme elevation and hyponatraemia was 5 of 11 patients (45.5%). From these patterns of clinical symptoms and laboratory findings, *Legionella* pneumonia seemed difficult to predict in these patients.

The usefulness of the six-point scoring system proposed by Fiumefreddo et al. [11] in predicting *Legionella* pneumonia was validated by Haubitz et al. [12]. They showed that a score of ≥ 5 had very high specificity of 99.0% (95% CI, 98.4–99.4) and high positive predictive value of 17.4% (95% CI, 5.0–38.8), whereas a score of < 2 had high sensitivity of 94.4% (95% CI, 81.3–99.3) and high negative predictive value of 99.6% (95% CI, 98.6–100). In this study, at a cut-off value of ≥ 2 points, the sensitivity of this scoring system was 54.5% (6/11) for non-*L. pneumophila* serogroup 1 patients and 95.7% (22/23) for *L. pneumophila* serogroup 1 patients. This finding indicated that we can rule out *Legionella* pneumonia due to *L. pneumophila* serogroup 1 by using this scoring system, whereas about half of patients with *Legionella* pneumonia due to non-*L. pneumophila* serogroup 1 could have been misdiagnosed by this scoring system. Namely, *Legionella* pneumonia due to non-*L. pneumophila* serogroup 1 cannot be ruled out by the six-point scoring system.

Yu et al. reported that consolidation and GGO were the main CT findings in 23 *Legionella* pneumonia patients and almost all patients (82.6%) had non-segmental distribution [26]. Sakai et al. showed that among 38 patients, consolidation plus GGO was seen in 35 (92.1%), pleural effusion was seen in 23 (60.5%) and ipsilateral hilar and/or mediastinal lymphadenopathy was seen in 17 (44.7%). They also indicated that sharply demarcated peribronchovascular foci of consolidation intermingled with GGO was a significant finding in *Legionella* pneumonia compared with *S. pneumoniae* pneumonia [27]. Similarly, our study showed that consolidation (72.7%) and GGO (81.8%) were common radiologic findings that were seen in 63.6% of patients. However, findings of pleural effusion (27.3%) and lymphadenopathy (27.3%) were relatively less common in our study than in the previous reports.

*Legionella* pneumonia due to non-*L. pneumophila* serogroup 1 may vary in severity from mild or moderate, which could be treated in an outpatient setting, to severe, which needs ICU admission and has poor prognosis. Only a few of these patients showed specific symptoms of *Legionella* pneumonia, such as neurologic abnormalities, myalgia or arthralgia, headache, diarrhoea and nausea or vomiting. In addition, abnormalities in laboratory tests, such as liver enzyme elevation and hyponatraemia, were not usual. Therefore, the six-point scoring system for *Legionella* pneumonia might not be useful for predicting cases due to non-*L. pneumophila* serogroup 1. Although this scoring system did not include radiologic findings, some previous reports and the present study showed that a combination of consolidation and GGO was a significant finding that might suggest the aetiology of pneumonia as *Legionella*. Therefore, in CAP patients showing consolidation plus GGO on chest CT and those with resistance to β-lactam antibiotic therapy, performing sputum culture for *Legionella* species on WYO-α or BCYE-α medium might be helpful. Furthermore, we should consider administration of antibiotics that are effective for *Legionella* species despite a negative *Legionella* urinary antigen test.

This study had some limitations. First, the study was retrospective and included a small number of patients. Second, in all cases due to non-*L. pneumophila* serogroup 1, *Legionella* species was identified by culture, which is not routine for all CAP patients; therefore, there might have been bias. Nevertheless, these results were important in providing data on the clinical characteristics of *Legionella* pneumonia due to non-*L. pneumophila* serogroup 1 and the usefulness of the six-point scoring system for predicting *Legionella* pneumonia due to non-*L. pneumophila* serogroup 1.

## Conclusions

In conclusion, most cases of *Legionella* pneumonia due to non-*L. pneumophila* serogroup 1 in this series presented as mild to moderate in severity. The six-point scoring system was not useful in predicting such cases. Therefore, urinary antigen test to detect non-*L. pneumophila* serogroup 1 would be expected in the future.

## Additional file

Additional file 1: Table S1. The six-point scoring system in patients with *Legionella* pneumonia due to *L. pneumophila* serogroup 1. (DOCX 19 kb)

## Abbreviations

Alb: albumin
BCYE-α: buffered charcoal yeast extract-α
BUN: blood urea nitrogen
CAP: community-acquired pneumonia
COPD: chronic obstructive pulmonary disease
CPK: creatine phosphokinase
Cr: creatinine
CRP: C-reactive protein
CT: computed tomography
CURB-65: confusion, urea > 7 mmol/L, respiratory rate ≥ 30 breaths/min, low blood pressure (systolic < 90 mmHg or diastolic ≤ 60 mmHg) and age ≥ 65 years
GGO: ground-glass opacity
Ht: haematocrit
ICU: intensive care unit
IQR: interquartile range
LDH: lactate dehydrogenase
Na: sodium
Plt: platelet
PSI: Pneumonia Severity Index
WBC: white blood cell
WYO-α: Wadowsky-Yee-Okuda-α
