# Supplementary material for: Legionella pneumonia due to non-Legionella pneumophila serogroup 1: usefulness of the six-point scoring system
Source: BMC Pulm Med. 2017 Dec 16;17:211. doi: 10.1186/s12890-017-0559-3 (PMC5732474; doi:10.1186/s12890-017-0559-3)
Supplement: Additional file 1: Table S1. — The six-point scoring system in patients with Legionella pneumonia due to L. pneumophila serogroup 1. (DOCX 19 kb) [file 12890_2017_559_MOESM1_ESM.docx]

Table S1. The six-point score in patients with *Legionella* pneumonia due to *L. pneumophila* serogroup 1

|  | CRP  >187 mg/L | Na <133 mmol/L | Temperature >39.4 °C | Plt  <171 × 10^9^/L | LDH  >225 IU/L | Dry cough | Total score |
| --- | --- | --- | --- | --- | --- | --- | --- |
| 1 | + | + | − | − | + | − | 3 |
| 2 | + | − | − | + | + | − | 3 |
| 3 | − | − | − | − | + | − | 1 |
| 4 | + | − | − | − | + | + | 3 |
| 5 | − | − | − | + | + | − | 2 |
| 6 | + | + | − | + | + | − | 4 |
| 7 | + | − | + | + | − | + | 4 |
| 8 | − | − | − | + | + | − | 2 |
| 9 | − | − | − | + | + | − | 2 |
| 10 | + | + | + | + | + | − | 5 |
| 11 | − | + | + | + | − | − | 3 |
| 12 | − | + | − | + | + | − | 3 |
| 13 | + | + | + | + | + | − | 5 |
| 14 | + | − | − | − | + | − | 2 |
| 15 | + | − | − | − | + | + | 3 |
| 16 | + | − | + | + | + | − | 4 |
| 17 | + | + | + | + | + | + | 6 |
| 18 | + | − | − | − | + | − | 2 |
| 19 | − | − | − | + | + | − | 2 |
| 20 | + | − | − | − | + | − | 2 |
| 21 | + | − | − | − | + | − | 2 |
| 22 | + | − | − | + | − | − | 2 |
| 23 | + | + | − | − | + | − | 3 |

*Abbreviations*: *CRP* C-reactive protein; *LDH* lactate dehydrogenase; *Plt* platelet
